# Long-term participation in community group exercise improves lower extremity muscle strength and delays age-related declines in walking speed and physical function in older adults

**DOI:** 10.1186/s11556-021-00260-2

**Published:** 2021-05-28

**Authors:** Chisato Hayashi, Soshiro Ogata, Tadashi Okano, Hiromitsu Toyoda, Sonoe Mashino

**Affiliations:** 1 Research Institute of Nursing Care for People and Community, University of Hyogo, 13-71 Kitaoji-cho, Akashi, Hyogo 673-8588 Japan; 2 Department of Preventive Medicine and Epidemiology, National Cerebral and Cardiovascular Center, 6-1 Kishibeshinmachi, Suita, Osaka, 564-8565 Japan; 3 Department of Orthopaedic Surgery, Osaka City University Graduate School of Medicine, 1-4-3 Asahi-machi, Abeno-ku, Osaka-City, Osaka, 545-8585 Japan

**Keywords:** Group exercises, Community-dwelling older adults, Lower extremity muscle strength, TUG time, Motor fitness scale

## Abstract

**Background:**

The effects of group exercise on the physical function of community-dwelling older adults remain unclear. The changes in lower extremity muscle strength, timed up and go (TUG) time, and the motor fitness scale (MFS), over time, among older adults who expressed a willingness to participate in community-based physical exercise groups, were determined using multilevel modelling.

**Methods:**

We analyzed data of 2407 older adults between April 2010 and December 2019 from the registry of physical tests of community-based physical exercise groups. We conducted a retrospective cohort study to assess the effect of physical exercise on lower extremity muscle strength, TUG time, and MFS scores. The durations of the exercises were evaluated by frequency of physical test’s participate.

**Results:**

A deterioration in lower extremity muscle strength was found in the short-term participant group only. However, in the mid-term and long-term participation groups, lower extremity muscle strength showed a trend of improvement. The TUG time and the MFS score were negatively correlated with increasing age in both groups divided by the duration of participation. However, there was a slower rate of deterioration in the long-term participation group.

**Discussion:**

Lower extremity muscle strength, TUG time, and MFS scores decline with increasing age and there were differences in the slope of deterioration that depended on the duration of participation in community-based group exercise.

**Conclusion:**

Participation in group exercise improved lower extremity muscle strength, TUG time, and MFS scores of older adults living in a community. The positive effects of group exercise were dependent on long-term participation.

**Supplementary Information:**

The online version contains supplementary material available at 10.1186/s11556-021-00260-2.

## Introduction

In 2019, the Japanese population aged over 65-years was estimated to be 35.88 million, and the aging rate was 28.4% [1]. In 2006, local governments, nationwide, started implementing long-term care prevention programs [2, 3], and in recent years, frailty prevention, which addresses the mental and physical deterioration associated with aging, has been attracting attention.

Maintenance of physical activity of older adults (age, > 65 years) leads to physical, mental, and social well-being, and ultimately, an extension of a healthy life expectancy. Participation in group activities such as club sports for adults are popular and have reported not only physical but also psychological and social benefits [4]. A recent study found that exercise with others has important health benefits regardless of the total frequency of exercise [5].

A study that examined 120 subjects (initial age, 46 to 78 years) suggested that the change in leg muscle strength was directly related to the change in muscle mass, and the longitudinal decline rates in knee extensor muscle strength were, on average, 14% per decade [6]. In addition, the annual decline in muscle strength ranged from 1.4 to 2.5% in healthy men (initial mean age, 65.4 ± 4.2 years) [7]. Lower extremity muscle strength declines faster than upper body muscle strength [8]; low muscle strength rather than muscle mass is associated with poor physical functioning in older adults [9]; and osteoporosis in older adults [10]. However, recent data from longitudinal studies on aging indicate that maintaining or gaining muscle mass does not prevent aging-related declines in muscle strength, and muscle weakness is independently associated with physical disability and mortality [11].

Although many studies have been conducted on physical performance in older adults, little is known about the effects of community-based group exercise on the change in physical performance. As older adults show an age-related decline in physical performance, the effect of activity is difficult to determine. In our study, we focused on “lively 100-years-old physical exercise”, which is a community group exercise for residents. This program was developed by doctors and physiotherapists from the Kochi Prefecture in 2003. The exercise may be performed while sitting on a chair or holding onto a chair for support to ensure safety of the participants. This type of group exercise, using light weights, is popular in the Japanese community.

This study aimed to determine the association between participation in community-based group exercise and physical performance in older adults, using multilevel modeling (MLM) which can model longitudinal changes in physical performance [12].

## Methods

### Study design and participants

We conducted a retrospective cohort study on older adults who expressed a willingness to participate in independent community-based physical exercise groups. In this study, we analysed data from the registry of physical tests between April 2010 and December 2019 of community-based physical exercise groups in Sumoto City, located in the central part of Awaji Island, Hyogo, Japan. In 2019, there were 85 small sub-groups and a total of 2407 participating in the “lively 100-years-old physical exercise” program. This study received approval from the Ethics Committee of the University of Hyogo (No.2019F21).

### Data

#### Exposure

The 40-min program consisted of stretching exercises, and seven types of muscle strengthening exercises [13]. Muscle strengthening exercises included lifting both arms up, lifting both arms to the side, getting up from the chair, knee extension exercises, knee lift exercises, lateral leg lifting exercises, and standing hip extension exercises. Weights were attached to the limbs and could be increased to 2.2 kg over 10 steps [13]. If the muscle pain persisted for > 1 week, the weights were reduced, and then increased again, by the participants, once the pain had subsided. In terms of when to change to a higher weight was determined by the participants themselves. Exercises were conducted once a week. However, the decision to participate was left to the individual.

#### Outcome

The physical fitness measurements of the participants were included for analysis. The participants were informed of the date and time of the physical fitness tests in advance. We assessed weight (kg), knee extension muscle strength (kgf), and TUG time (sec). Knee extension muscle strength (kgf) was adjusted to body weight (%). As an indicator of lower extremity muscle strength, the strength of the knee extensors was measured using a hand-held dynamometer (μTas F-1; Anima Co., Tokyo, Japan) (Supplemental image 1) [14]. The reproducibility and validity of determining the knee extension force, using a hand-held dynamometer, has been described in young healthy subjects [14] and hemiplegic patients [15]. Measurements were recorded continuously by one trained physical therapist. No preparatory exercises were performed. The maximum value of two consecutive measurements was registered for further analysis. With the participants seated in a chair, the length of the belt was adjusted so that the knee joint was at 90° when a force was applied. Measurements were taken with a fixation belt worn over the two lateral digits from the outer edge of the tibia (Supplemental image 2). TUG was measured as a direct physical performance test. The TUG test is a reliable, cost-effective, safe, and time-efficient way to evaluate overall functional mobility [16] and measures the time taken to stand up from a chair, to walk and turn around at a mark 3 m ahead, then back to being fully seated in the chair again.

In addition, we used the motor fitness scale (MFS) score to evaluate the physical ability of the participants, based on mobility that was equivalent to the direct measurement of physical performance among older residents who had improved levels of health status and functioning [17]. The highest total score attainable was 14 points, with higher scores indicating better physical performance. The MFS displays a high internal consistency (α = 0.92) and test-retest reliability (individual correlation [ICC] = 0.92) [18]; it comprises three subscales including mobility, muscle strength, and balance.

#### Covariates

Covariates that were controlled included age, sex, the frailty index (activities of daily living, motor skills, low nutrition, dysphagia, cognitive function, depression, and home-boundedness), and the Kihon Checklist scores (KCL) [19]. This checklist included a self-administered questionnaire consisting of a total of 25 questions.

#### Duration of exercises

At a “lively 100-years-old physical exercise” session, older adults were required to participate in physical fitness tests every 4 months during the first year, and thereafter, once annually. The total number of physical fitness tests per participant ranged from 1 to 13 times. The 25th percentile was three times and the 75th percentile was seven times. Therefore, in this analysis, all participants were divided into three groups according to the total number of participations and completed physical tests. Participants who took the tests < 3 times were placed in a short-term participation group, those who took the tests four to six times were placed in the mid-term participation group, and those who took the tests seven to 13 times were placed in the long-term participation group.

### Statistical analyses

All statistical analyses were conducted using R version 3.6.3 (Vienna, Austria), with the lme4 package [20] to fit the mixed-effects models [21]. Mixed-effects models can adequately adjust for missing data as an outcome variable, by the missing at random (MAR) assumption. Maximum likelihood methods were used for the analysis of missing data because the pattern of the missing data was not missing completely at random (MCAR). MLM is an extension of regression that allows for simultaneous estimation of fixed and random effects and is additionally robust for unbalanced data (i.e., missing observations) [22–24]. The t-test and the chi-square test were used to compare the baseline data. The level of significance was set at *p* <  0.05.

MLM was used to identify the physical performance slope difference between groups of different ages. Age was centred by the mean age of all participants. Centering changes an estimated intercept score in models, but does not influence estimated regression coefficients in the model. Intercept scores can be interpreted as the predicted value of outcome where age was mean age and the other predictors were zero except. We used the model to estimate the mean at each point in the reference grid for each age from 65 to 90 years old. Marginal means were estimated as equally weighted means of these predictions at specified margins.

We investigated the associations between the participation level and changes in lower extremity muscle strength, TUG time, and MFS using the following three models: Model 1 was an unadjusted model with a random slope model. Model 2 was adjusted for age, sex, frailty index (depression, home-boundedness, dysphagia, poor nutrition, cognitive status) with a random slope model.

## Results

### Baseline demographic characteristics

A comparison of baseline demographic characteristics is shown in Table 1. The mean total duration of the physical tests from baseline was 2.35 years (SD = 2.51), the mean duration of the short-term group was 0.75 years (SD = 1.21), the mean duration of the mid-term group was 3.81 years (SD = 1.60), and the mean duration of the long-term group was 6.44 years (SD = 1.19). The mean age of all the participants was 74.3 years (SD = 8.0), the mean body weight was 52.5 kg (SD = 9.4), the mean knee extension strength was 19.0 kgf (SD = 11.6), the mean lower extremity muscle strength was 50.7% (SD = 19.4), the mean TUG time was 7.6 s (SD = 3.4), and the mean MFS score was 10.6 points (SD = 3.5). In total, 17.0% of the sample was male. The total number of missing values for age was 4, sex was 6, muscle strength was 183, TUG was 126, and MFS was 43. The difference in the mean age between groups was not significant at baseline (*p* = 0.13). The lower extremity muscle strength was significantly lower in the long-term participant group than in the short-term participant group (*p* <  0.001). However, TUG time was significantly faster in the long-term participant group than in the short-term participant group (*p* <  0.001). In addition, the difference in mean MFS score was significantly higher in the long-term participant group than in the short-term participant group (*p* <  0.001).

**Table 1:** Baseline characteristics of participants

	All					Male				Female			
	Total	short-term	mid-term	long-term	*P*-Value	short-term	mid-term	long-term	*P*-Value	short-term	mid-term	long-term	*P*-Value
N	2570	1553	636	381		277	96	63		1275	540	318	
**Duration from baseline physical test (year)**													
mean (sd)	2.35 (2.51)	0.75 (1.21)	3.81 (1.60)	6.44 (1.19)	<.001	0.69 (1.06)	3.51 (1.49)	6.35 (1.38)	<.001	0.76 (1.24)	3.87 (1.62)	6.46 (1.16)	<.001
**Age (year)**													
mean (sd)	74.3 (8.0)	74.5 (8.5)	74.2 (7.2)	73.6 (6.7)	0.130	74.3 (8.3)	74.5 (6.7)	71.8 (7.2)	0.064	74.5 (8.6)	74.1 (7.3)	74.0 (6.6)	0.418
**Age75**^a^ **(year)**													
mean (sd)	-0.1 (1.6)	-0.1 (1.7)	-0.2 (1.4)	-0.3 (1.3)	0.130	-0.1 (1.7)	-0.1 (1.3)	-0.6 (1.4)	0.064	-0.1 (1.7)	-0.2 (1.5)	-0.2 (1.3)	0.418
**Body weight (kg)**													
mean (sd)	52.5 (9.4)	53.2 (10.1)	52.6 (9.0)	53.7 (8.7)	0.142	61.8 (10.1)	60.9 (9.2)	63.5 (7.3)	0.259	51.3 (9.0)	51.2 (8.1)	51.8 (7.6)	0.513
**Knee extension strength (kgf)**													
mean (sd)	32.0 (11.6)	28.0 (11.2)	25.7 (9.7)	23.2 (9.1)	<.001	33.3 (13.0)	33.5 (12.0)	32.9 (11.8)	0.950	26.9 (10.4)	24.3 (8.6)	21.3 (7.1)	<.001
**Lower extremity muscle strength**^b^ **(%)**													
mean (sd)	50.7 (19.4)	53.2 (20.6)	49.2 (17.7)	42.9 (14.5)	<.001	53.9 (20.2)	55.3 (18.8)	52.0 (18.4)	0.586	53.0 (20.7)	48.2 (17.3)	41.1 (12.9)	<.001
**TUG time**^c^ **(sec)**													
mean (sd)	7.6 (3.4)	7.8 (3.9)	7.3 (2.3)	7.3 (2.1)	<.001	8.2 (4.7)	7.0 (2.4)	6.7 (1.6)	0.006	7.7 (3.7)	7.3 (2.2)	7.4 (2.1)	0.018
**Motor fitness scale (point)**													
mean (sd)	10.6 (3.5)	10.3 (3.7)	10.9 (3.1)	11.2 (3.1)	<.001	10.6 (3.3)	11.0 (3.2)	12.1 (2.4)	0.003	10.3 (3.8)	10.9 (3.1)	11.0 (3.2)	<.001

^a^Age 75 was value after centering for 75 years old

^b^Lower extremity muscle strength adjusted to body weight (%)

^c^TUG time was obtained timed up and go test

### Multilevel linear regression models

First, we estimated the intra-ICC by null models as a fixed effect only. ICC is used to give a sense of how much variance is explained by a random effect in MLM. For muscle strength, over half of the variance was between individuals. In the TUG test, over 60% of the variance was in TUG time of individuals. For the MFS, approximately 80% of the variance in the score was between individuals. That is, 50–80% of the variance, not explained by the linear effect of age, was attributable to person-to-person differences. Moreover, the unexplained or residual variance was less for the model that included person-specific slopes (trajectories over time).

**Fig. 1:**
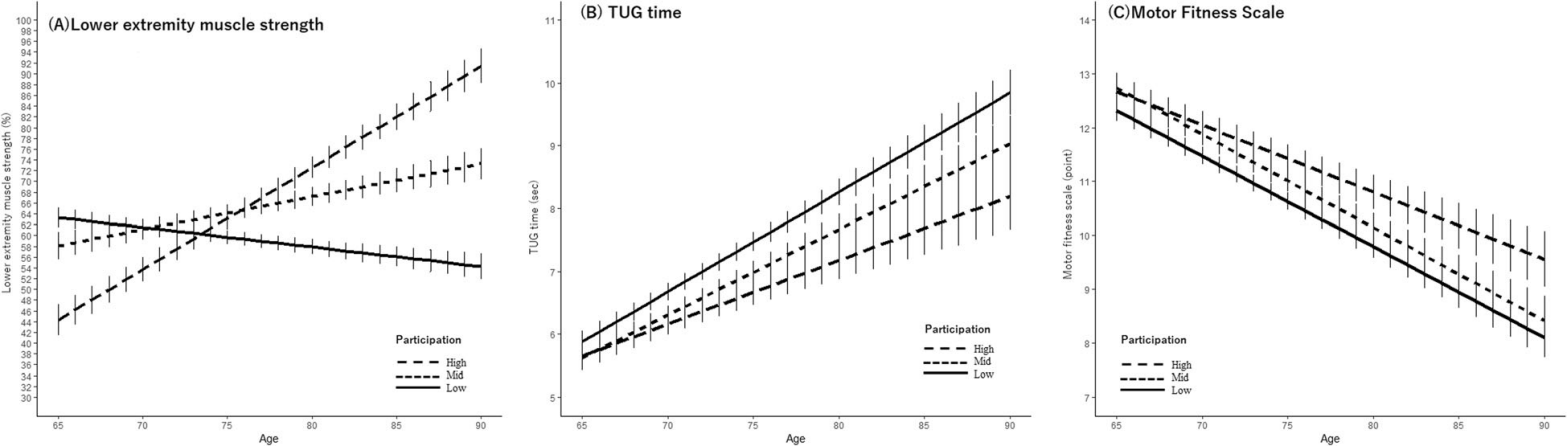
Slopes of changes and estimated marginal means (95% confidence interval) of participants in community-dwelling group exercises. There were three panels: **a** weight-adjusted knee muscle strength; **b** timed up and go; **c** motor fitness scale. The slopes and estimated marginal means were obtained from linear mixed models. Bar lines show 95% confidence intervals for the estimated marginal means at each 5-year point. Figure 1 and Supplemental Table 1 correspond

We specifically wanted to know if the trajectory over time differed for the short-term, mid-term, and long-term participation groups. This translated into a fixed-effects interaction between age and groups. We examined the effect of age on lower extremity muscle strength (Fig. 1a). A slope of deterioration in lower extremity muscle strength was found only in the short-term participant group. However, in the mid-term and long-term participation groups, the slope showed a trend of improvement. Next, we examined the effect of age on TUG time (Fig. 1b). The TUG time declined with increasing age in both groups divided by the duration of participation. Finally, we examined the effect of age on MFS score (Fig. 1c). The MFS score also declined with increasing age in both groups divided by the duration of participation. The TUG time and the MFS scores worsened in all groups. However, there was a slower rate of deterioration in the long-term participation group. For every 5-year increase in age, the long-term participation group reported faster TUG time and higher MFS scores at all periods compared with those who discontinued the exercises earlier. The estimated marginal means are shown in Supplemental Table 1.

Table 2 shows regression coefficients obtained from the multilevel linear regression models. Model 1 was an unadjusted model. In Model 2, we controlled for the frequency of participation, sex, depression, frailty index (home-bound, dysphagia, poor nutrition, cognitive status). In Model 2, lower extremity muscle strength intercept scores of 59.88% were determined and declined significantly at a rate of − 1.82% for every additional year of age (*p* <  0.001). On average, a TUG time intercept score of 7.06 s was determined and then declined significantly by 0.79 s for every additional year of age (*p* <  0.001). Moreover, the MFS intercept scores were 11.14 points and declined significantly by − 0.84 points for every additional year of age (*p* <  0.001). One way to judge a model is to compare the estimated means to the observed means to determine how accurately they are represented by the model. This excellent fit of the estimated marginal means to the observed data supports the hypothesis that the change across age was linear. The interaction term for frequency of participation and age was significant, indicating that the gradient of age varied according to the frequency of participation because the interaction for age and frequency of participation was significant (Table 2).

**Table 2:** Regression coefficients obtained from the multilevel linear regression models

	Lower extremity muscle strength ^4^	*p*-value	TUG time ^5^	*p*-value	Motor fitness scale	*p*-value
	*N* = 2383 (total^1^ = 7816)		*N* = 2400 (total^1^ = 7857)		*N* = 2411 (total^1^ = 7931)	
**Model1**^2^						
Intercept	59.18 (58.34, 60.02)	< 0.001	7.23 (7.13, 7.33)	< 0.001	10.79 (10.67, 10.90)	< 0.001
slope changes of age	3.16 (2.50, 3.83)	< 0.001	0.76 (0.70, 0.82)	< 0.001	−0.90 (−0.97, −0.84)	< 0.001
**Model2**^3^						
Intercept	59.88 (58.59, 61.16)	< 0.001	7.06 (6.90, 7.22)	< 0.001	11.14 (10.97, 11.31)	< 0.001
slope changes of age	− 1.82 (− 2.51, − 1.12)	< 0.001	0.79 (0.70, 0.88)	< 0.001	− 0.84 (− 0.94, − 0.75)	< 0.001
Participation						
short-term	0 (Reference)		0 (Reference)		0 (Reference)	
mid-term	4.46 (2.67, 6.25)	< 0.001	− 0.48 (− 0.70, − 0.27)	< 0.001	0.38 (0.14, 0.63)	0.002
long-term	3.46 (1.34, 5.57)	0.001	− 0.80 (−1.04, − 0.56)	< 0.001	0.80 (0.51, 1.08)	< 0.001
Interaction terms between age and participation						
age:short-term	0 (Reference)		0 (Reference)		0 (Reference)	
age:mid-term	4.85 (3.68, 6.05)	< 0.001	−0.11(− 0.25, 0.03)	0.128	− 0.02 (− 0.17, 0.13)	0.721
age:long-term	11.25 (10.00, 12.52)	< 0.001	− 0.28 (− 0.44, − 0.13)	< 0.001	0.22 (0.07, 0.38)	0.006
Covariates						
sex	3.33 (1.48, 5.19)	< 0.001	0.07(− 0.13, 0.27)	0.477	0.09(− 0.14, 0.33)	0.427
depression	− 0.90 (− 1.25, − 0.55)	< 0.001	0.14 (0.09, 0.19)	< 0.001	− 0.23 (− 0.28, − 0.19)	< 0.001
home-bound	− 1.97 (−2.82, − 1.13)	< 0.001	0.44 (0.32, 0.56)	< 0.001	− 0.45 (− 0.55, − 0.35)	< 0.001
dysphagia	−0.40(− 0.90, 0.10)	0.119	0.01(− 0.06, 0.08)	0.774	− 0.16 (− 0.22, − 0.10)	< 0.001
poor nutrition	1.32 (0.50, 2.14)	0.001	0.09(− 0.03, 0.21)	0.129	0(− 0.09, 0.10)	0.928
cognitive status	− 0.82 (− 1.39, − 0.25)	0.005	0.14 (0.05, 0.22)	0.001	−0.13 (− 0.20, − 0.06)	< 0.001

^1^ Records due to repeated physical test. ^2^ Model 1 was unadjusted

^3^ Model 2 was adjusted for frequency of participation, sex, depression, home-bound, oral status, poor nutrition and cognitive status

^4^ Lower extremity muscle strength adjusted to body weight (%) ^5^ TUG time obtained by timed up and go test

## Discussion

To the best of our knowledge, this is the first study to clarify the relationship between longer-term participation in group exercise and the positive effect in older adults. Notably, this is the first MLM analysis to be applied to a physical assessment of the participation of the “lively 100-years-old physical exercise” program. This study not only found that lower extremity muscle strength, TUG time, and MFS scores declined with age, but differences in slopes depended on the duration of participation in group exercises in the community were also determined.

A deterioration in lower extremity muscle strength was only observed for the short-term participation group. However, in the mid-term and long-term participation groups, the slope showed an increasing trend. This study showed that participation in group exercise may improve the lower extremity muscle strength of older adults living in the community, even after removing the effect of age. There was a substantial change in the slope of lower extremity muscle strength, with the largest slope observed for the long-term participation group. In this study, the slope of weight loss was almost the same for the duration of participation as shown in the appendix, however, the muscle strength increased in the middle- and long-term participation groups*.* This suggested that rather than an increase in muscle mass, there was an increase in muscle force output. Random effects represent between-subject effects. Adding the random slopes did not significantly change the estimates for the fixed effects.

It is known that among community-dwelling older adults, objective measures of lower extremity function have been highly predictive of subsequent disability [25]. A recent study found that age-related loss of muscle strength was only partially explained by the reduction in muscle mass and that other physiologic factors explained the muscle weakness in older adults [26]. Previous research suggests that muscle weakness in older adults is more likely as a result of impairments in central neural activation and/or reductions in the intrinsic force-generating capacity of skeletal muscle [27–29]. This study revealed that participation in group exercise had a positive effect on the knee muscle strength even when age-related changes were removed. A systematic review found that predominantly balance and functional training reduced falls compared with an inactive control group in older adults living in the community [30]. Lower extremity muscle power was no better than knee-extension torque or handgrip in the early identification of poor mobility, defined either as a walking speed of < 0.8 m/s or the inability to walk at least 1 km without difficulty [31]. It has also been shown to be useful in the evaluation of group exercises in the community. Further investigations are needed to determine how improvements in muscle strengthening, with active exercise, affects older adults.

In terms of TUG time, the change worsened in all groups. In terms of MFS scores, the change also worsened in all groups. However, there was a slower slope of deterioration in the long-term participation group. A previous study reported that MFS scores of social educational activities increased significantly from 11.67 ± 2.54 points to 11.84 ± 2.54 points (*p* = 0.043) with a high number of sessions (≥7 times: twice or more a month) after 6 months, and from 11.58 ± 2.70 points to 11.79 ± 2.67 points (*p* = 0.007) in all participants [32]. An examination of the cut-off point, combined with the results of the MFS scores, should be considered in the future.

In this study, group exercise increased lower extremity muscle strength in the long-term and mid-term participation groups, but TUG time and MFS score did not improve and weakened with a deterioration in age. Previous research reported that in frail adults, resistance training increased strength and TUG time [33]. However, in stronger community-dwelling adults, resistance training further increased strength but did not affect TUG time [34]. It has been shown that when the isometric knee extensor strength was 0.5 kgf/kg (50%) or higher, the effect of muscle strength on gait parameters was small (mean age, 75.0 years old) [35]. The estimated muscle strength intercept scores in participants of this study was 59.88% which may have influenced the results.

Despite accounting for age-related declines, group exercise among older adults living in the community was found to have a positive impact on the duration of participation. The “lively 100-years-old physical exercise” which is gaining popularity in Japan, was discontinued in this city between April and May 2020 due to the spread of a COVID-19; however, 90% of the groups had resumed by August. Community-based initiatives in which older adults proactively get together and exercise in their neighbourhoods have been shown to delay the functional decline associated with aging, which should motivate the government, older adults, and their families to work on long-term care prevention. A systematic review reported that only exercise programs that were conducted in groups were effective in preventing frailty [36]. Exercises can influence the muscle during aging and should be emphasized as part of a lifestyle essential to healthy aging.

This study has limitations. First, because this study used secondary data, we could not determine other potentially related factors, such as history of diseases. Second, as the reasons for participants quitting the exercise group were unknown, we could not assess the status of their final physical tests. Third, as this is a retrospective cohort study with secondary data, we are unable to determine the causal relationship. Fourth, we measured kgf as the unit of knee extension muscle strength. When converted to Newton (N), which is used as an International System of Units System (SI), the value is kgf × 9.80665. Kg is used as a unit of body weight and is easy to understand for community-dwelling older adults in Japan, and grip strength was explained in kgf. Therefore, kgf/weight (%) was used in this study. Fifth, since the values are measured by a hand-held dynamometer that considers the fixation system using a belt, they are lower than those measured by an isokinetic muscle force measuring device. However, it is versatile in that it can compensate for the problems associated with manual immobilization [37]. There were no participants who only participated in physical fitness tests. It is possible that participants who disliked the physical fitness tests may have not attended the tests, but all participants attended the first test and the effect on the results is considered to be small.

## Conclusion

The lower extremity muscle strength intercept scores of 59.88% were determined and changed by approximately − 2% for every additional year of age (*p* <  0.001). On average, a TUG time intercept score of 7.06 s was determined and changed by approximately 1 s for every additional year of age (*p* <  0.001). Moreover, the MFS intercept scores were 11.14 points and then changed by approximately − 1 points for every additional year of age (*p* <  0.001). These finding create a basis for further studies in this area. This study not only found that lower extremity muscle strength, TUG time, and MFS scores decline with age, but also that differences in the slope depended on the duration of participation in group exercise in the community. Long-term participation in community group exercise improves lower extremity muscle strength and delays age-related declines in walking speed and physical function in older adults, despite the effects of aging.

### Supplementary Information

**Additional file 1: Supplemental Table 1.** Estimated marginal means of lower extremity muscle strength, TUG time and motor fitness scale obtained by linear mixed models.

**Additional file 2: Supplemental image 1.** A hand-held dynamometer (μTas F-1; Anima Co., Tokyo, Japan).

**Additional file 3: Supplemental image 2.** An image of the dynamometer set up.

## Data Availability

The datasets used during the current study are available from the corresponding author on reasonable request.

## Appendix

Slopes of changes and estimated marginal means (95% confidence interval) of participants in community-dwelling group exercises. There were three panels: (D) weight. Bar lines show 95% confidence intervals for the estimated marginal means at each 5-year point.
