# Supplementary figures and images for: Long-term participation in community group exercise improves lower extremity muscle strength and delays age-related declines in walking speed and physical function in older adults
Source: Eur Rev Aging Phys Act. 2021 May 28;18:6. doi: 10.1186/s11556-021-00260-2 (PMC8161349; doi:10.1186/s11556-021-00260-2)

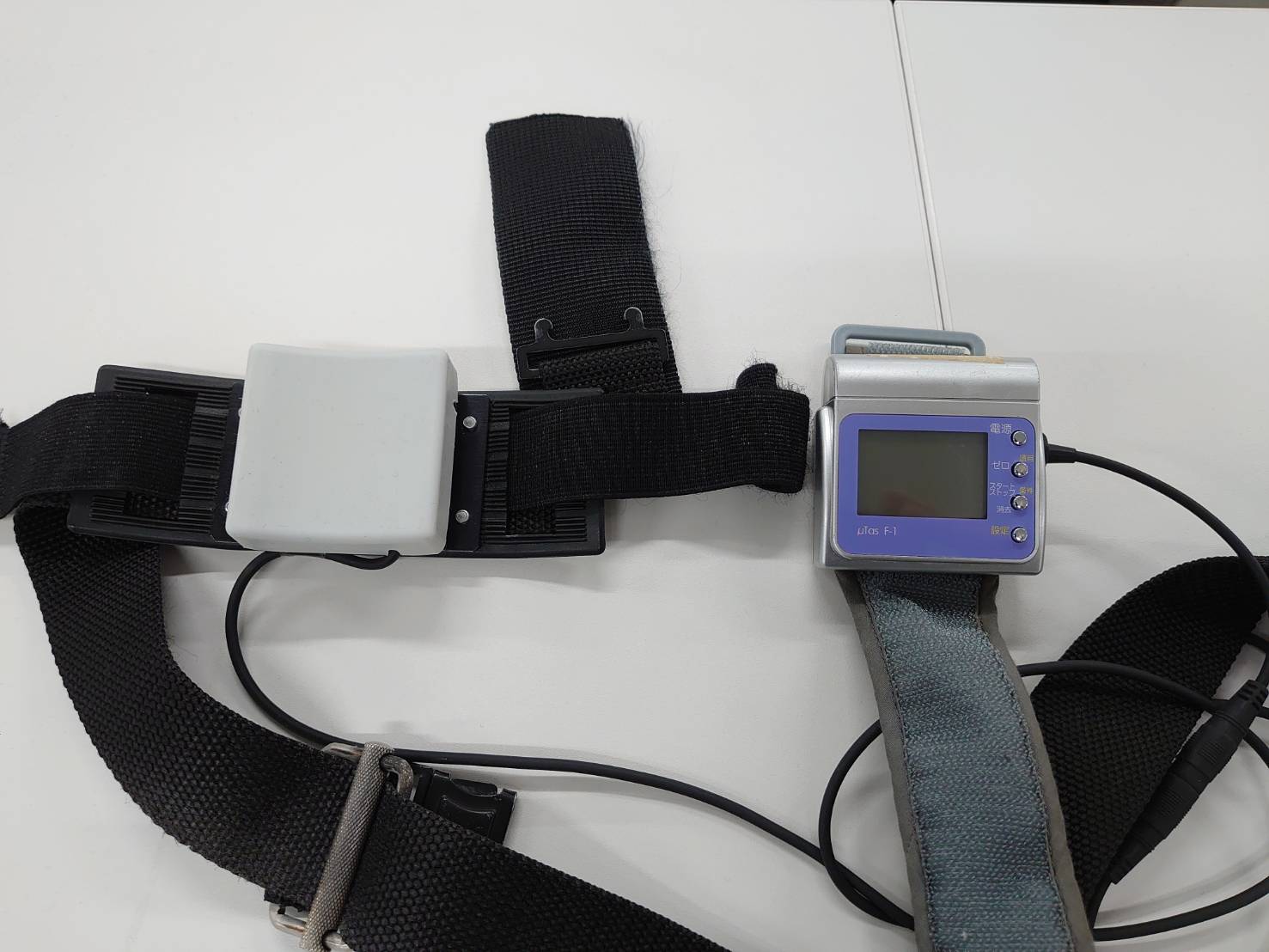

Supplement: Supplementary file 2 — Additional file 2: Supplemental image 1. A hand-held dynamometer (μTas F-1; Anima Co., Tokyo, Japan). [file 11556_2021_260_MOESM2_ESM.jpg]

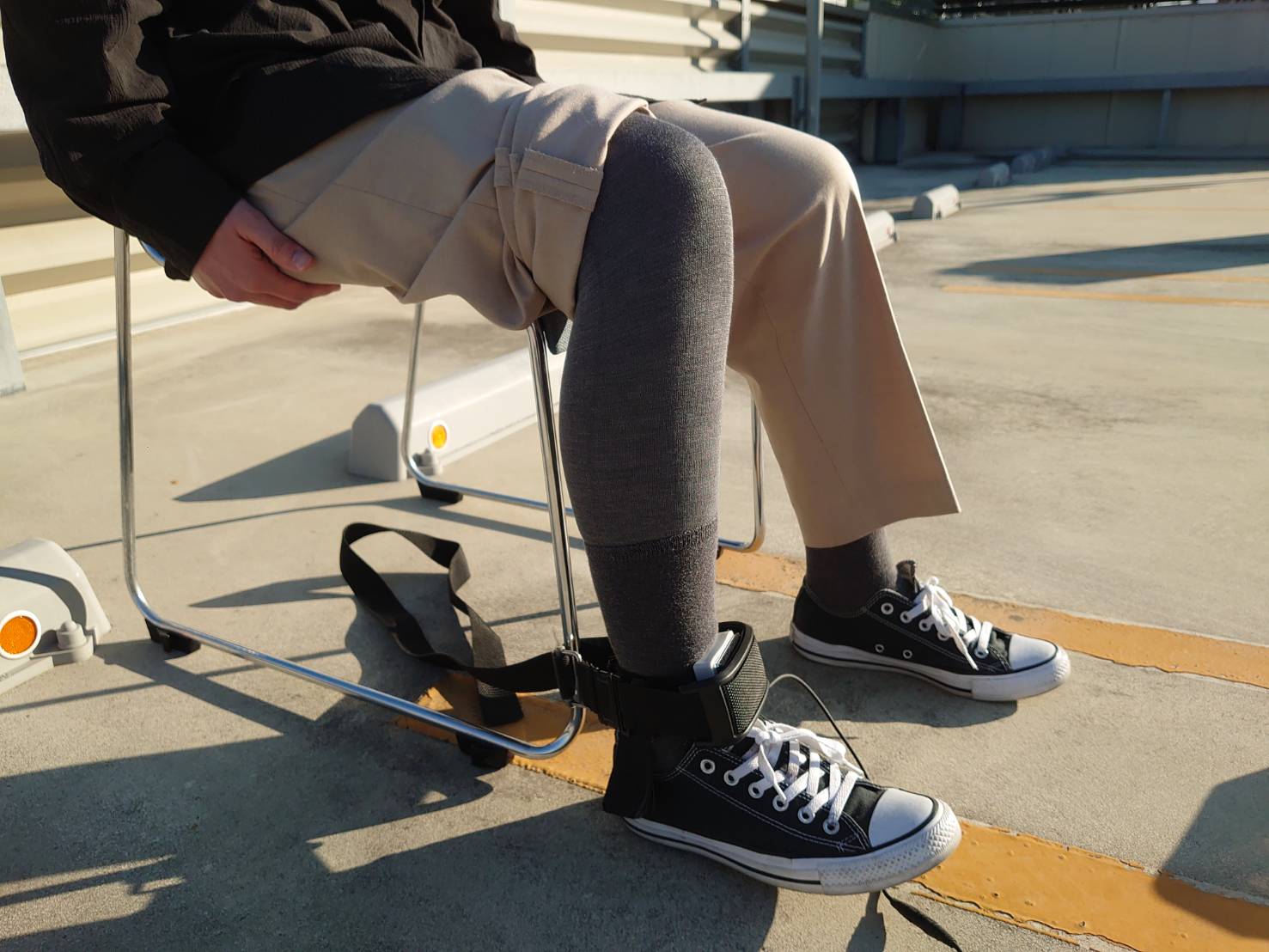

Supplement: Supplementary file 3 — Additional file 3: Supplemental image 2. An image of the dynamometer set up. [file 11556_2021_260_MOESM3_ESM.jpg]
